# Obstetric ultrasound training programmes for midwives: A scoping review

**DOI:** 10.4102/hsag.v28i0.2163

**Published:** 2023-01-27

**Authors:** Yasmin Casmod, Susan J. Armstrong

**Affiliations:** 1 Department of Nursing Education, Faculty of Health Sciences, University of the Witwatersrand, Johannesburg, South Africa; 2 Department of Medical Imaging and Radiation Sciences, Faculty of Health Sciences, University of Johannesburg, Johannesburg, South Africa

**Keywords:** midwives, obstetric ultrasound, antenatal care, education, training

## Abstract

**Background:**

Antenatal care is essential for all expectant mothers and assists in reducing maternal mortality rates thus addressing the Sustainable Development Goal 3. Obstetric ultrasound complements antenatal care and is used in pregnancy to identify and monitor high-risk pregnancies. However, disparities exist and in low- and middle-income countries, ultrasound services are not readily available. This contributes to maternal and neonatal morbidity and mortality within these populations. Short ultrasound training programmes for midwives can be beneficial in alleviating some of the challenges experienced.

**Aim:**

The aim of this scoping review was to identify global ultrasound education programmes for midwives.

**Method:**

Articles containing suitable keywords were retrieved from databases suitable to nursing, education and ultrasound. Themes were developed based on the articles included in the review.

**Results:**

A total of 238 articles were identified, and after the duplicates and irrelevant studies were removed, 22 articles were included. Articles were analysed and discussed under the identified themes and categories.

**Conclusion:**

It is essential that sufficient training is provided to medical professionals performing obstetric ultrasound so that adequate and safe care is offered to expectant mothers. With the introduction of ultrasound in low-resource settings, the knowledge of safety and competencies required to operate the equipment necessitate adequate training. Developed programmes have been found to meet the demands of the ever-changing workforce and allow for midwives to perform focused obstetric ultrasound examinations.

**Contribution:**

This scoping review highlighted ultrasound training programmes for midwives and provided guidance on the development of future midwifery ultrasound training programmes.

## Introduction

Appropriate and suitable antenatal care services provide screening for pregnant women and offer clinicians valuable information on the wellbeing of both the mother and the unborn foetus. Antenatal care is significant in reducing obstetric complications, thus enhancing the services provided by trained healthcare workers and assisting in decreasing the mortality and morbidity rates (Holmlund et al. 2019). Obstetric ultrasound, a significant component of antenatal care, is a non-invasive imaging modality (Dornhofer et al. 2020) that has been identified as an important and valuable tool used routinely in pregnancy (Vinayak et al. 2017). Ultrasound imaging produces real-time images of the developing foetus (Fullerton et al. 2019), and it has been suggested that every pregnant woman should have at least one ultrasound during pregnancy (World Health Organization [WHO] 2016) to determine viability, accurately determine the gestational age, determine the number of foetuses, estimate the due date, determine the location of the pregnancy, locate the placenta and quantify the amniotic fluid volume. Furthermore, obstetric ultrasound offers valuable information by identifying high-risk pregnancies and further enhances the care provided. This assists in providing the appropriate diagnosis, reduces the complications and interventions at delivery and decreases foetal mortality and morbidity rates (Holmlund et al. 2017).

Globally obstetric ultrasound images are acquired and interpreted by a wide range of medical professionals which include sonographers, obstetricians, midwives, nurses and radiologists (Sousa & Corning-Davis 2019). Owing to the shortage of trained healthcare professionals able to offer a diagnostic ultrasound service, many pregnant women in low- and middle-income countries do not have access to ultrasound imaging even though the value of obstetric ultrasound has been extensively documented (Vinayak et al. 2017).

Ultrasound training and availability vary within and between countries, and all pregnant women are not afforded equal opportunities because of the existing disparities. Within South Africa, sonographers are trained through higher education learning institutions and, once qualified, are able to conduct a wide variety of ultrasound examinations; however, there are no formal or standardised ultrasound training programmes available for midwives. Midwives are the primary providers of antenatal care and are often the first contact for many expectant women and thus play an important role in the care of expectant mothers and their babies. Thus, the introduction of ultrasound training programmes for all healthcare workers including midwives is warranted. The safety and accuracy of ultrasound in pregnancy have been established if used correctly by trained healthcare professionals and offer valuable information in diagnosing and managing pregnant patients.

The aim of this scoping review was to identify and report on worldwide ultrasound education programmes for midwives.

## Methods

The Joanna Briggs guidelines on the methodology applicable to scoping reviews were followed. The scoping review was guided by answering the following research question: What obstetric ultrasound education do midwives receive globally? A further sub-question was asked: What is included in an ultrasound curriculum for midwives?

The following steps were applied:

- Identify the research question
- Identify relevant studies
- Study selection
- Charting the data
- Collating, summarising and reporting the results
- A scoping review was conducted and identification of relevant studies.

A methodical electronic search was undertaken to recognise the available literature on midwifery ultrasound training programmes globally between January 2010 and June 2020. The keywords used to search databases were ‘midwives’, ‘obstetric ultrasound’ and ‘education/training’. These keywords were grouped together, allowing for an all-inclusive search. Databases searched were Scopus, PubMed, Cumulative Index to Nursing and Allied Health Literature (CINAHL – hosted within Ebscohost), Education Resources Information Centre (ERIC), and ProQuest.

### Study selection

**FIGURE 1:**
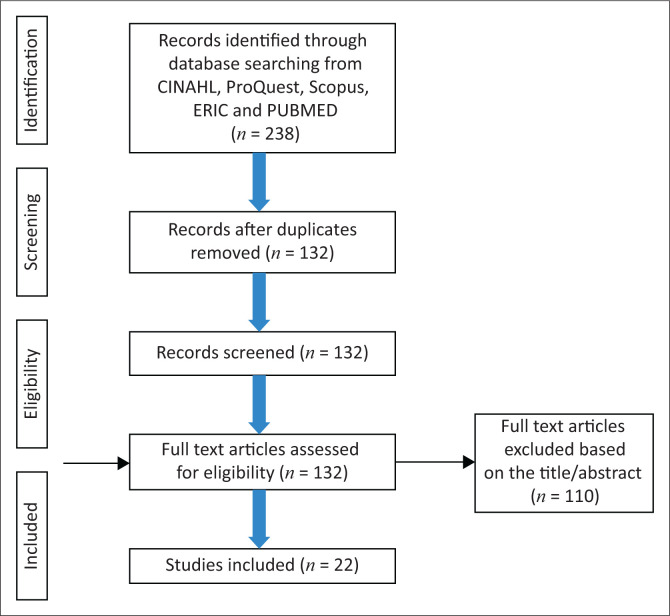
A PRISMA diagram for the search process and inclusion.

Ultrasound training for midwives was strengthened after the year 2010, and thus English literature that was published between January 2010 and June 2020 was included. Furthermore, this allowed for an identification of the current practices. Records that were identified were screened for duplication, and all duplicates were removed from the initial search. All full-text records were screened for eligibility and relevance to the research question based on the title or abstract. Subsequently, all full-text records remaining were further reviewed and screened for suitability. This process is demonstrated in Figure 1.

### Charting the data

A Microsoft word document was used to gather applicable data from the studies selected. As demonstrated in Table 1, each category was completed in a separate column and included the following:

- The authors
- The aim and/or purpose
- The population and setting
- The design and findings.

**TABLE 1:** Studies included in the scoping review.

No.	Author, title	Aim and/or purpose	Population and setting	Design and findings
1	Vinayak, S. & Brownie, S., 2018, ‘Collaborative task-sharing to enhance the Point-Of-Care Ultrasound (POCUS) access among expectant women in Kenya: The role of midwife sonographers’, *Journal of Interprofessional Care* 32(5), 641–644. https://doi.org/10.1080/13561820.2018.1470499	To train midwives basic Point of Care Ultrasound (POCUS).	Midwives, radiologists and sonographers, Kenya	Design and development research Midwives identified as the suitable population for inter-professional task shifting because of the shortage of sonographers Train midwives to perform basic obstetric ultrasound for early detection and referral of high-risk pregnancies.
2	Swanson, J.O., Kawooya, M.G., Swanson, D.L., Hippe, D.S., Dungu-Matovu, P. & Nathan, R., 2014, ‘The diagnostic impact of limited, screening obstetric ultrasound when performed by midwives in rural Uganda’, *Journal of Perinatology* 34(7), 508–512. https://doi.org/10.1038/jp.2014.54	To evaluate the diagnostic impact of limited obstetric ultrasound in identifying high-risk pregnancies when used as a screening tool by midwives in rural Uganda.	Midwives, Rural Uganda	Design and development research High-risk pregnancies can be identified with obstetric ultrasound The cost and size of the equipment have been identified as a barrier Midwives are able to provide a limited obstetric service after receiving training
3	Åhman, A., Edvardsson, K., Kidanto, H.L., Ngarina, M., Small, R. & Mogren, I., 2018, ‘“Without ultrasound you can’t reach the best decision” – Midwives’ experiences and views of the role of ultrasound in maternity care in Dar Es Salaam, Tanzania’, *Sexual and Reproductive Healthcare* 15(November 2017), 28–34. https://doi.org/10.1016/j.srhc.2017.11.007	To explore Tanzanian midwives’ experiences and views of the role of obstetric ultrasound in relation to the clinical management of pregnancy.	Midwives, Dar Es Salaam	Qualitative study Pregnancy management is improved with the use of ultrasound. Because of the shortage of equipment and skilled healthcare workers ultrasound services are not always available. Training needs to be provided to enhance practical skills
4	Dornhofer, K., Farhat, A., Guan, K., Parker, E., Dornhofer, K., Farhat, A., Guan, K., Parker, E., Kong, C., Kim, D. et al., 2020, ‘Evaluation of a point-of-care ultrasound curriculum taught by medical students for physicians, nurses, and midwives in rural Indonesia’, *Journal of Clinical Ultrasound* 48(3), 145–151. https://doi.org/10.1002/jcu.22809	Medical students are able to effectively teach POCUS to nurses, physicians, and midwives	Physicians, Nurses and Midwives, Rural Indonesia	Prospective observational study Ultrasound skills can be taught to healthcare workers through adequate training programmes. Ultrasound machines are compact and can be easily obtained. Lectures, didactics, and hands-on training methods were used to teach the necessary skills.
5	Dolo, O., Clack, A., Gibson, H., Lewis, N. & Southall, D.P., 2016, ‘Training of midwives in advanced obstetrics in Liberia’, *Bulletin of the World Health Organization* 94(5), 383–387. https://doi.org/10.2471/blt.15.160473	The aim was to provide advanced obstetric training to midwives.	Liberia Midwives	Pilot Project Training midwives in advanced obstetrics in an effort to address staff shortages is warranted and demonstrates effectiveness
6	Baj, N., Dubbins, P. & Evans, J.A., 2015, ‘Obstetric ultrasound education for the developing world: A learning partnership with the World Federation for Ultrasound in Medicine and Biology’, *Ultrasound* 23(1), 53–58. https://doi.org/10.1177/1742271X14566848	To provide support for trainees in both urban and rural sites.	Uganda	Pilot project Evaluated using close-ended questions. High-risk pregnancies can be identified with the timely use of ultrasound to facilitate referrals.
7	Vinayak, S., Sande, J., Nisenbaum, H. & Nolsoe, P., 2017, ‘Training midwives to perform basic obstetric point-of-care ultrasound in rural areas using a tablet platform and mobile phone transmission technology – A WFUMB COE project’, *Ultrasound in Medicine and Biology* 43(10), 2125–2132. https://doi.org/10.1016/j.ultrasmedbio.2017.05.024	To determine the accuracy of images and reports generated by trained midwives. To evaluate the performance of a portable ultrasound scanner. The use of teleradiology	Midwives, Kenya	Pilot project Prospective cross-sectional study. Barriers in low-resource settings have been identified as a cause of perinatal mortality and morbidity in low-resource settings Training midwives in low-income countries to perform focused obstetric ultrasounds is a practical option and will address staff shortages.
8	Sousa, M.F. & Corning-Davis, B., 2019, ‘Building capacity to provide maternal health care in an indigenous Guatemalan Community through ultrasound and skills training’, *Journal of Radiology Nursing* 38(2), 123–130. https://doi.org/10.1016/j.jradnu.2019.03.002	Aim to bring ultrasound services and skills in a local clinic in Guatemala.	Midwives, nurses, radiographers and Medical Officers, Guatemala	Pilot project Ultrasound is needed to detect high-risk pregnancies
9	Bentley, S., Hexom, B. & Nelson, B.P., 2015, ‘Evaluation of an obstetric ultrasound curriculum for midwives in Liberia’, *Journal of Ultrasound in Medicine* 34(9), 1563–1568. https://doi.org/10.7863/ultra.15.14.08017	To evaluate the effectiveness of a 1-week obstetric ultrasound training course	Midwives, Liberia	Quantitative prospective observational study Course provided adequate knowledge retention The use of ultrasound in pregnancy enhances clinical diagnosis and midwives are able to learn the skill.
10	Holmlund, S., Ntaganira, J., Edvardsson, K., Lan, P.T., Sengoma, J.P.S., Ahman, A. et al., 2017, ‘Improved maternity care if midwives learn to perform ultrasound: A qualitative study of Rwandan midwives’ experiences and views of obstetric ultrasound’, *Global Health Action* 10(1), 1350451. https://doi.org/10.1080/16549716.2017.1350451	To explore Rwandan midwives’ experiences and views on the role of obstetric ultrasound	Midwives, Rwanda	Qualitative study Training of midwives to perform obstetric ultrasound will improve the access for Rwandan women and address the unequal access.
11	Kozuki, N., Mullany, L.C., Khatry, S., Ghimire, R.K., Paudel, S., Blakemore, K. et al., 2016, ‘Accuracy of home-based ultrasonographic diagnosis of obstetric risk factors by primary-level health care workers in rural Nepal’, *Obstetrics and Gynecology* 128(3), 604–612. https://doi.org/10.1097/AOG.0000000000001558	To assess the feasibility of ultrasound task shifting	Auxiliary nurse-midwives, Rural Nepal	Task sharing has the potential to increase access to ultrasound services. Primary health care workers with limited training can accurately conduct ultrasound examinations in limited resource settings, to establish peripartum risk factors.
12	Shaw-Battista, J., Young-Lin, N., Bearman, S., Dau, K. & Vargas, J., 2015, ‘Interprofessional obstetric ultrasound education: Successful development of online learning modules; case-based seminars; and skills labs for registered and advanced practice nurses, midwives, physicians, and trainees’, *Journal of Midwifery and Women’s Health* 60(6), 727–734. https://doi.org/10.1111/jmwh.12395	To describe the foundations of the developed course and review the challenges and solutions in obstetric ultrasound	Healthcare workers, USA	Review Develop interprofessional education The low cost of machines can assist in overcoming identified barriers. Obstetric ultrasound training should be standardised for midwives
13	Barnfield, L., Bamfo, J. & Norman, L., 2019, ‘Should midwives learn to scan for presentation? Findings from a large survey of midwives in the UK’, *British Journal of Midwifery* 27(5), 305–311. https://doi.org/10.12968/bjom.2019.27.5.305	To assess local practices and attitudes towards midwives scanning for presentation.	Midwives and Midwifery students, UK	Online survey Availability of the service can be increased because of the decrease in the price of ultrasound machines High sensitivity and specificity with short training programmes for midwives. Midwifery practice can be enhanced if midwives are trained to scan for foetal presentation.
14	Shah, S., Santos, N., Kisa, R., Maxwell, O.M., Mulowooza, J., Walker, D. et al., 2020, ‘Efficacy of an ultrasound training program for nurse midwives to assess high-risk conditions at labor triage in rural Uganda’, *PLoS One* 15(6 June), 1–15. https://doi.org/10.1371/journal.pone.0235269	To identify the impact of POCUS on six obstetric complications	Midwives and physicians, Eastern Uganda	Mixed methods Training courses should be implemented to identify high-risk pregnancies. These will provide confidence and improved ultrasound skills. Technological advances make the use of ultrasound feasible.
15	Holmlund, S., Ntaganira, J., Edvardsson, K., Lan, P.T., Sengoma, J.P.S., Kidanto, H.L. et al., 2018, ‘Health professionals’ experiences and views on obstetric ultrasound in Rwanda: A crosssectional study’, *PLoS One* 13(12), 1–20. https://doi.org/10.1371/journal.pone.0208387	The aim of this study was to investigate Rwandan health professionals’ experiences and views of obstetric ultrasound in relation to clinical management, resources and skills.	Obstetricians, physicians, midwives and nurses, Rwanda	Prospective, Qualitative study Implementation of a pregnancy dating service will be beneficial. Further training in ultrasound is required. Short courses for healthcare workers such as midwives with no previous experience can be beneficial and can improve maternity services to allow timeous referrals Barriers to providing an ultrasound service exist because of a lack of trained professionals and the cost of equipment.
16	Kimberly, H.H., Murray, A., Mennicke, M., Liteplo, A., Lew, J., Bohan, J.S. et al., 2010, ‘Focused maternal ultrasound by midwives in rural Zambia’, *Ultrasound in Medicine and Biology* 36(8), 1267–1272. https://doi.org/10.1016/j.ultrasmedbio.2010.05.017	Introduce an ultrasound training programme for midwives to reduce maternal and new-born mortality.	Midwives, Zambia	Pilot project Midwives can be trained to use portable ultrasound machines to perform basic obstetric ultrasound examinations and will compensate for the shortage of obstetricians.
17	McClure, E.M., Nathan, R.O., Saleem, S., Esamai, F., Garces, A., Chomba, E. et al., 2014, ‘First look: A cluster-randomized trial of ultrasound to improve pregnancy outcomes in low income country settings’, *BMC Pregnancy and Childbirth* 14(1), 1–9. https://doi.org/10.1186/1471-2393-14-73	Aims to prove that antenatal ultrasound screening will improve pregnancy outcomes.	Nurses, midwives and clinical officers, Five low-income countries	Cluster randomised trial Barriers identified; however, ultrasound machines are decreasing in cost and becoming increasingly available and thus pregnancy outcomes can be improved.
18	Holmlund, S., Lan, P.T., Edvardsson, K., Phuc, H.D., Ntaganira, J., Small, R. et al., 2019, ‘Health professionals’ experiences and views on obstetric ultrasound in Vietnam: A regional, cross-sectional study’, *BMJ Open* 9(9), 1–12. https://doi.org/10.1136/bmjopen-2019-031761	The aim of this study was to explore Vietnamese health professionals’ experiences and views of obstetric ultrasound in relation to clinical management, resources and skills.	Obstetricians/gynaecologists, Midwives Hanoi, Vietnam	Cross-sectional questionnaire Need for additional training of ultrasound users to improve pregnancy outcomes
19	Kinnevey, C., Kawooya, M., Tumwesigye, T., Douglas, D. & Sams, S., 2016, ‘Addressing obstetrical challenges at 12 rural Ugandan health facilities: Findings from an international ultrasound and skills development training for midwives in Uganda’, *International Journal of MCH and AIDS (IJMA)* 5(1), 46–52. https://doi.org/10.21106/ijma.106	To assess the perspectives of rural Ugandan clinics on current barriers to antenatal care to identify deficiencies in prenatal services and anticipated new demands with the addition of ultrasound	Midwives, Rural Uganda	Prospective survey Lack of ultrasound services is a significant barrier in patient care Equipping facilities with training and ultrasound machines are a cost-effective way to improve maternal and child health
		To assess rural Ugandan midwives’ perspectives on the perceived challenges they would face by adding ultrasound to their health facilities and identifying knowledge deficiencies in the management of prenatal conditions.		
20	Boamah, E.A., Asante, K.P., Ae-Ngibise, K.A., Kinney, P.L., Jack, D.W., Manu, G. et al., 2014, ‘Gestational age assessment in the Ghana Randomized Air Pollution and Health Study (GRAPHS): Ultrasound capacity building, fetal biometry protocol development, and ongoing quality control’, *JMIR Research Protocols* 3(4), e77. https://doi.org/10.2196/resprot.3797	To optimise gestational age assessment. Secondary, training and capacity building	Midwives, Ghana	Design and Development Research To determine gestational age, appropriate training and methods to obtain foetal biometry should be provided.
21	Edvardsson, K. Ntaganira, J., Ahman, A., Sengoma, J.P.S., Small, R. & Mogren, I., 2016, ‘Physicians’ experiences and views on the role of obstetric ultrasound in rural and urban Rwanda: A qualitative study’, *Tropical Medicine and International Health* 21(7), 895–906. https://doi.org/10.1111/tmi.12718	To explore Rwandan physicians’ experiences and views on the role of obstetric ultrasound.	Physicians, Rwanda	Exploratory qualitative study Ultrasound is regarded as an important aspect of antenatal care and enhances pregnancy management; thus, training midwives to perform ultrasound examinations is necessary.
22	Fullerton, J., Butler, M., Aman, C. & Reid, T., 2019, ‘Global competencies for midwives: External cephalic version; ultrasonography, and tobacco cessation intervention’, *Women and Birth* 32(3), e413–e420. https://doi.org/10.1016/j.wombi.2018.08.166	The aim is to review the process about three specific clinical practices.	Africa, America and Europe	Review Various training methods reported, and once adequately trained, midwives can accurately perform ultrasound scans, thus providing reassurance.

Data collection, summary and reporting of findings.

A total of 22 studies were included. Findings were contextualised by year of publication, article distribution in journals, geographical distribution and author collaborations. Furthermore, through the process of thematic analysis, emerging themes and categories were identified.

### Ethical considerations

Ethical Clearance Certificate (R14/49) was provided by the Human Research Ethics Committee (Medical), University of the Witwatersrand.

## Results

The primary search produced 238 articles. After removing the duplicates, 132 articles were screened for eligibility and 110 records were eliminated. Thus, 22 studies were included as per the study protocol. Four studies were part of the larger multinational cross-country ultrasound study (CROCUS), which explored the experiences and views of midwives and obstetricians in low- and middle-income countries with regard to the use of ultrasound and pregnancy management. Seven papers were pilot and prospective observational/cross-sectional studies, respectively. Two studies were reviews. One study was an online survey, and one was a cluster randomised trial.

**FIGURE 2:**
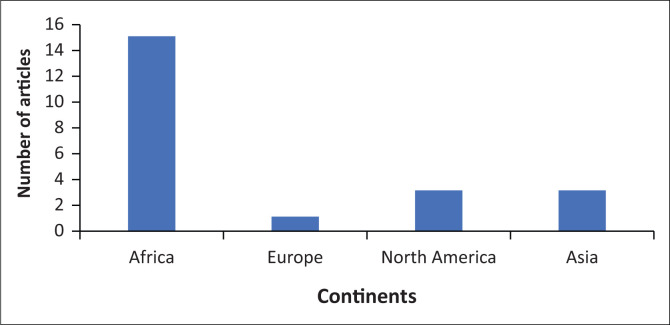
Geographical distribution of included articles.

As demonstrated in Figure 2, 15 (68%) of the studies included were from Africa, while 3 (14%) of the studies were from North America and Asia, respectively, and only 1 (4%) study was from Europe.

**FIGURE 3:**
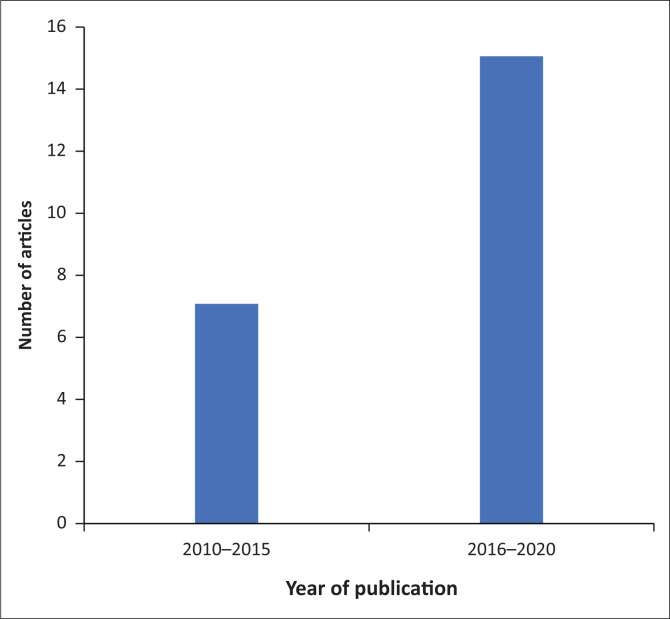
Articles by year of publication.

As demonstrated in Figure 3, an increase in article publication was noted between 2016 and 2020 with 68% of the articles published during this time frame. Thirty-two percent of the articles included were published between 2010 and 2015.

Sixty-four percent (*n* = 14) of the articles included in the study are from the following journals, one from each: *Journal of Inter-professional care, Journal of Perinatology, Journal of Sexual & reproductive health, Bulletin of the World Health Organization, Journal of Global Health Action, Journal of Obstetrics & Gynecology, Journal of Midwifery & Women’s Health, British Journal of Midwifery, Journal of Pregnancy & Childbirth, British Medical Journal, International Journal of Maternal & Child Health, Journal of Medical Internet Research, Journal of Tropical Medicine & International Health and Journal of Women & Birth.* There were 27% (*n* = 6) of the articles published in ultrasound and radiology journals, while 9% (*n* = 2) of the articles were published in *PLOS ONE*. All included articles were co-authored.

## Discussion

Once the data were collected, the process of data analysis began. As demonstrated in Table 2, through the process of thematic analysis, the following themes and sub-themes were identified.

**TABLE 2:** Themes and categories.

Themes	Sub-themes
1. Barriers	Lack of equipment Lack of trained professionals Lack of training programmes Lack of skills
2. Task sharing	Task shifting Role extension
3. Improving antenatal care	Early detections Management of high-risk pregnancies Timeous referrals
4. Ultrasound training programmes	Basic obstetric ultrasound Skills retention and mentoring

Fifteen articles identified barriers to the use of ultrasound. Task sharing and including ultrasound training by extending the role of midwives were identified in all studies. All articles also identified that the use of obstetric ultrasound improves antenatal care. Ultrasound training methods for midwives were included in all of the studies.

### Theme 1: Barriers

Trained users are able to interpret ultrasound findings and provide an adequate diagnosis, thus facilitating referrals and improving pregnancy management and outcomes. However, the most significant challenge and barrier identified with the use of obstetric ultrasound is the lack of education and training programmes specifically for midwives (Dornhofer et al. 2020). The lack of this specific training programme is brought about by insufficient trained and skilled healthcare professionals (Bentley, Hexom & Nelson 2015; Holmlund et al. 2018). Ultrasound is operator dependent, and ultrasound used by inadequately trained healthcare professionals can lead to negative outcomes and false positives (Holmlund et al. 2018). Providing appropriate and adequate training to midwives is of utmost importance, as the production and interpretation of the ultrasound image are directly associated with the training received. Providing an adequate and timely diagnostic ultrasound service is known to improve pregnancy management and outcomes (Baj, Dubbins & Evans 2015); however, because of the shortage of trained healthcare professionals, the ability to provide this service in all settings is compromised (Swanson et al. 2014). Because of these factors identified, ultrasound services are not readily available in all settings. Thus, introducing training programmes allows for healthcare professionals to offer equal antenatal services to all expectant mothers.

In the past the complexity, cost (McClure et al. 2014) and size of the ultrasound equipment were identified as a challenge; however, with the advances in the development of ultrasound equipment, the affordability, the portable compact size as well as the robust nature of this technology, introducing ultrasound in all settings has become a viable option (Kimberly et al. 2010). Furthermore, the image quality on these portable compact machines is of an acceptable standard, and these units if used correctly by trained healthcare workers are capable of providing images of a suitable diagnostic quality.

### Theme 2: Task sharing

Task sharing is a safe and applicable method used to reallocate tasks from highly trained professionals to professionals trained in a short period with lower qualifications (Vinayak & Brownie 2018). Task sharing addresses gaps in the workforce among healthcare workers (Vinayak & Brownie 2018) and can increase the accessibility of ultrasound services because of the lack of trained medical professionals (Kozuki et al. 2016), thus addressing the unequal distribution and availability of this service. Furthermore, referrals can be reduced if all midwives are equipped with this important and significant skill thus reducing the burden to an already strained healthcare system. Offering standardised training and support will result in successful task sharing.

Midwives are frontline healthcare workers that offer antenatal services and are thus the ideal population to offer ultrasound training to. It has been demonstrated that midwives after having received appropriate training are able to use portable ultrasound machines to perform basic obstetric ultrasound scans and incorporate ultrasound into their daily practice (Kimberly et al. 2010). Furthermore, it has been demonstrated that midwives are able to accurately determine gestational age if suitable training is provided (Boamah et al. 2014). Thus, training midwives to perform obstetric ultrasound is a suitable (Dolo et al. 2016) and cost-effective method to further enhance maternal and child health (Kinnevey et al. 2016).

### Theme 3: Improving antenatal care

Differences in maternal mortality and morbidity rates between high- and low-income countries are attributed to the lack of antenatal care and the limited access to healthcare facilities (McClure et al. 2014). An identified priority for low- and middle-income countries include timely identification and diagnosis of pregnancy-related complications (Kozuki et al. 2016). Obstetric ultrasound is an essential element of antenatal care in the developed world and can improve pregnancy management in high-risk pregnancies (Åhman et al. 2018) and reduce perinatal mortality and morbidity rates if introduced routinely in the developing world (Holmlund et al. 2018). Furthermore, it is essential that sufficiently trained healthcare workers provide antenatal services to patients to assist in reducing maternal and neonatal mortality rates (Holmlund et al. 2017).

A basic obstetric ultrasound scan is identified as an important tool in the management of pregnancies (Edvardsson et al. 2016) and further improves the accuracy of physical examinations. The routine use of obstetric ultrasound is able to identify pregnancy complications and possible high-risk pregnancies that could be missed with a clinical examination (Swanson et al. 2014). Furthermore, obstetric ultrasound increases diagnostic accuracy (Kozuki et al. 2016) and offers appropriate diagnosis and interventions (Barnfield, Bamfo & Norman 2019; Holmlund et al. 2017), thus allowing for timeous referrals. Induced labour can be reduced with the use of ultrasound by providing accurate gestational age assessments (Holmlund et al. 2019). Ultrasound is encouraging, complements clinical assessments and enhances clinical decisions (Shaw-Battista et al. 2015). This allows for improved pregnancy management, adequate preparation for labour and birth and reduces the number of surprises for women and healthcare workers at birth (Kinnevey et al. 2016).

### Theme 4: Ultrasound training programmes

Ultrasound education and training programmes for midwives have been conducted using various training methods (Fullerton et al. 2019) and time frames. These include didactics, hands-on training, distance online training programmes and formal site-based training programmes. Furthermore, written learning objectives, a skills checklist and the supervision of practical competencies were included (Shaw-Battista et al. 2015). Training programmes varied, however, included aspects of equipment maintenance, ultrasound physics, normal and pathological anatomy of the mother and the foetus, image storage, appropriate reporting methods and basic communication skills between the patient and the healthcare worker (Kinnevey et al. 2016).

Majority of the training programmes includes training midwives to perform basic obstetric ultrasound scans. An important aspect of training programmes is the inclusion of the diagnostic capabilities and ethical responsibilities of the end user to ensure a diagnostic ultrasound service (Holmlund et al. 2019). Short focused ultrasound training programmes of varying duration ranging from 1 week to 6 months (Boamah et al. 2014) have demonstrated high sensitivity and specificity (Barnfield et al. 2019). Conducting practical assessments as part of training is strongly supported and confirms that midwives have obtained the skill and training required to practise independently thus providing this service to a greater population (Fullerton et al. 2019). Post-training assessment methods indicate that knowledge gained through the training programmes was retained, thus further indicating the success in training midwives. Furthermore, ongoing mentoring was identified as an important aspect for the sustainable training of midwives (Shah et al. 2020). Training programmes have demonstrated that midwives are able to develop self-confidence and skills after participating in a structured ultrasound training programme. Furthermore, midwives are able to execute the required task and identify high-risk pregnancies.

## Conclusion

Over recent years, the midwife’s role has advanced and grown substantially to keep up with societal demands, expectations and the everchanging workforce, by incorporating focused obstetric ultrasound training programmes for midwives (Shaw-Battista et al. 2015). Studies demonstrated that midwifery ultrasound services and tertiary led ultrasound services are comparable (Fullerton et al. 2019), and thus, the key to a successful training programme is standardisation.

## References

[CIT0001] Åhman, A., Edvardsson, K., Kidanto, H.L., Ngarina, M., Small, R. & Mogren, I., 2018, ‘“without ultrasound you can’t reach the best decision” – Midwives’ experiences and views of the role of ultrasound in maternity care in Dar Es Salaam, Tanzania’, *Sexual and Reproductive Healthcare* 15(November 2017), 28–34. 10.1016/j.srhc.2017.11.007 29389498

[CIT0002] Baj, N., Dubbins, P. & Evans, J.A., 2015, ‘Obstetric ultrasound education for the developing world: A learning partnership with the World Federation for Ultrasound in Medicine and Biology’, *Ultrasound* 23(1), 53–58. 10.1177/1742271X14566848 27433236 PMC4760565

[CIT0003] Barnfield, L., Bamfo, J. & Norman, L., 2019, ‘Should midwives learn to scan for presentation? Findings from a large survey of midwives in the UK’, *British Journal of Midwifery* 27(5), 305–311. 10.12968/bjom.2019.27.5.305

[CIT0004] Bentley, S., Hexom, B. & Nelson, B.P., 2015, ‘Evaluation of an obstetric ultrasound curriculum for midwives in Liberia’, *Journal of Ultrasound in Medicine* 34(9), 1563–1568. 10.7863/ultra.15.14.08017 26254155

[CIT0005] Boamah, E.A., Asante, K.P., Ae-Ngibise, K.A., Kinney, P.L., Jack, D.W., Manu, G. et al., 2014, ‘Gestational age assessment in the Ghana Randomized Air Pollution and Health Study (GRAPHS): Ultrasound capacity building, fetal biometry protocol development, and ongoing quality control’, *JMIR Research Protocols* 3(4), e77. 10.2196/resprot.3797 25525828 PMC4376157

[CIT0006] Dolo, O., Clack, A., Gibson, H., Lewis, N. & Southall, D.P., 2016, ‘Training of midwives in advanced obstetrics in Liberia’, *Bulletin of the World Health Organization* 94(5), 383–387. 10.2471/blt.15.160473 27147768 PMC4850529

[CIT0007] Dornhofer, K., Farhat, A., Guan, K., Parker, E., Kong, C., Kim, D. et al., 2020, ‘Evaluation of a point-of-care ultrasound curriculum taught by medical students for physicians, nurses, and midwives in rural Indonesia’, *Journal of Clinical Ultrasound* 48(3), 145–151. 10.1002/jcu.22809 31876301

[CIT0008] Edvardsson, K. Ntaganira, J., Ahman, A., Sengoma, J.P.S., Small, R. & Mogren, I., 2016, ‘Physicians’ experiences and views on the role of obstetric ultrasound in rural and urban Rwanda: A qualitative study’, *Tropical Medicine and International Health* 21(7), 895–906. 10.1111/tmi.12718 27125579

[CIT0009] Fullerton, J., Butler, M., Aman, C. & Reid, T., 2019, ‘Global competencies for midwives: External cephalic version; ultrasonography, and tobacco cessation intervention’, *Women and Birth* 32(3), e413–e420. 10.1016/j.wombi.2018.08.166 30174206

[CIT0010] Holmlund, S., Lan, P.T., Edvardsson, K., Phuc, H.D., Ntaganira, J., Small, R. et al., 2019, ‘Health professionals’ experiences and views on obstetric ultrasound in Vietnam: A regional, cross-sectional study’, *BMJ Open* 9(9), 1–12. 10.1136/bmjopen-2019-031761 PMC6773349 31548354

[CIT0011] Holmlund, S., Ntaganira, J., Edvardsson, K., Lan, P.T., Sengoma, J.P.S., Ahman, A. et al., 2017, ‘Improved maternity care if midwives learn to perform ultrasound: A qualitative study of Rwandan midwives’ experiences and views of obstetric ultrasound’, *Global Health Action* 10(1), 1350451. 10.1080/16549716.2017.1350451 28764602 PMC5645676

[CIT0012] Holmlund, S., Ntaganira, J., Edvardsson, K., Lan, P.T., Sengoma, J.P.S., Kidanto, H.L. et al., 2018, ‘Health professionals’ experiences and views on obstetric ultrasound in Rwanda: A crosssectional study’, *PLoS One* 13(12), 1–20. 10.1371/journal.pone.0208387 PMC6279039 30513102

[CIT0013] Kimberly, H.H., Murray, A., Mennicke, M., Liteplo, A., Lew, J., Bohan, J.S. et al., 2010, ‘Focused maternal ultrasound by midwives in rural Zambia’, *Ultrasound in Medicine and Biology* 36(8), 1267–1272. 10.1016/j.ultrasmedbio.2010.05.017 20691916

[CIT0014] Kinnevey, C., Kawooya, M., Tumwesigye, T., Douglas, D. & Sams, S., 2016, ‘Addressing obstetrical challenges at 12 rural Ugandan health facilities: Findings from an international ultrasound and skills development training for midwives in Uganda’, *International Journal of MCH and AIDS (IJMA)* 5(1), 46–52. 10.21106/ijma.106 28058192 PMC5187639

[CIT0015] Kozuki, N., Mullany, L.C., Khatry, S., Ghimire, R.K., Paudel, S., Blakemore, K. et al., 2016, ‘Accuracy of home-based ultrasonographic diagnosis of obstetric risk factors by primary-level health care workers in rural Nepal’, *Obstetrics and Gynecology* 128(3), 604–612. 10.1097/AOG.0000000000001558 27500343 PMC5028110

[CIT0016] McClure, E.M., Nathan, R.O., Saleem, S., Esamai, F., Garces, A., Chomba, E. et al., 2014, ‘First look: A cluster-randomized trial of ultrasound to improve pregnancy outcomes in low income country settings’, *BMC Pregnancy and Childbirth* 14(1), 1–9. 10.1186/1471-2393-14-73 24533878 PMC3996090

[CIT0017] Shah, S., Santos, N., Kisa, R., Maxwell, O.M., Mulowooza, J., Walker, D. et al., 2020, ‘Efficacy of an ultrasound training program for nurse midwives to assess high-risk conditions at labor triage in rural Uganda’, *PLoS One* 15(6 June), 1–15. 10.1371/journal.pone.0235269 PMC7326214 32603339

[CIT0018] Shaw-Battista, J., Young-Lin, N., Bearman, S., Dau, K. & Vargas, J., 2015, ‘Interprofessional obstetric ultrasound education: Successful development of online learning modules; case-based seminars; and skills labs for registered and advanced practice nurses, midwives, physicians, and trainees’, *Journal of Midwifery and Women’s Health* 60(6), 727–734. 10.1111/jmwh.12395 26769384

[CIT0019] Sousa, M.F. & Corning-Davis, B., 2019, ‘Building capacity to provide maternal health care in an indigenous Guatemalan Community through ultrasound and skills training’, *Journal of Radiology Nursing* 38(2), 123–130. 10.1016/j.jradnu.2019.03.002

[CIT0020] Swanson, J.O., Kawooya, M.G., Swanson, D.L., Hippe, D.S., Dungu-Matovu, P. & Nathan, R., 2014, ‘The diagnostic impact of limited, screening obstetric ultrasound when performed by midwives in rural Uganda’, *Journal of Perinatology* 34(7), 508–512. 10.1038/jp.2014.54 24699218

[CIT0021] Vinayak, S. & Brownie, S., 2018, ‘Collaborative task-sharing to enhance the Point-Of-Care Ultrasound (POCUS) access among expectant women in Kenya: The role of midwife sonographers’, *Journal of Interprofessional Care* 32(5), 641–644. 10.1080/13561820.2018.1470499 29746179

[CIT0022] Vinayak, S., Sande, J., Nisenbaum, H. & Nolsoe, P., 2017, ‘Training midwives to perform basic obstetric point-of-care ultrasound in rural areas using a tablet platform and mobile phone transmission technology – A WFUMB COE project’, *Ultrasound in Medicine and Biology* 43(10), 2125–2132. 10.1016/j.ultrasmedbio.2017.05.024 28716434

[CIT0023] World Health Organization, 2016, *WHO recommendations on antenatal care for a positive pregnancy experience*, World Health Organization, Geneva, Switzerland. 28079998

